# Supplementary material for: Investigation of Combined Cyclodextrin and Hydrogel Formulation for Ocular Delivery of Dexamethasone Acetate by Means of Experimental Designs
Source: Pharmaceutics. 2018 Dec 1;10(4):249. doi: 10.3390/pharmaceutics10040249 (PMC6320851; doi:10.3390/pharmaceutics10040249)
Supplement: Supplementary file 1 [file pharmaceutics-10-00249-s001.pdf]

pharmaceutics

MDPI

Supplementary Materials: Investigation of Combined Cyclodextrin and Hydrogel Formulation for Ocular Delivery of Dexamethasone Acetate by Means of Experimental Designs

Roseline Mazet, Luc Choisnard, Delphine Levilly, Denis Wouessidjewe and Annabelle Gèze

Table S1. Optimization of mixed gel based on HPβCD.

Experiments	Gel1: CELLUVISC®		Gel2: GEL-LARMES®		Gel3: VISMED®		HPβCD 600mg/mL with DXMa 10 mg/mL		Adjunction of DXMa (mg)	Osmolality (mOsm/Kg)	DXMa solubility (mg/mL)
	Theoretical proportion (%)	Real proportion (%)	Theoretical proportion (%)	Real proportion (%)	Theoretical proportion (%)	Real proportion (%)	Theoretical proportion (%)	Real proportion (%)			
1	70.0	69.9	0.0	0.0	0.0	0.0	30.0	30.1	10.676	364	2.657
2	0.0	0.0	70.0	69.8	0.0	0.0	30.0	30.2	10.811	399	2.625
3	0.0	0.0	0.0	0.0	70.0	69.4	30.0	30.6	10.228	243	2.992
4	0.0	0.0	0.0	0.0	0.0	0.0	100.0	100.0	10.154	765	10.912
5	35.0	35.0	35.0	35.3	0.0	0.0	30.0	29.8	11.148	368	2.743
14-1	23.3	23.5	23.3	22.8	23.3	23.7	30.0	30.0	11.598	324	2.861
6	35.0	35.2	0.0	0.0	35.0	34.8	30.0	30.0	10.274	293	2.735
7	35.0	35.0	0.0	0.0	0.0	0.0	65.0	65.0	10.122	494	6.375
8	0.0	0.0	35.0	35.9	35.0	34.7	30.0	29.5	10.078	312	2.725
9	0.0	0.0	35.0	35.1	0.0	0.0	65.0	64.9	10.014	521	6.195
10	132.9	0.0	0.0	0.0	35.0	35.1	65.0	64.9	10.356	407	6.292
14-2	23.3	23.5	23.3	23.7	23.3	23.3	30.0	29.4	10.018	321	2.677
11	0.0	0.0	23.3	22.7	23.3	24.2	53.3	53.1	10.964	391	5.118

12	23.3	24.2	0.0	0.0	23.3	23.1	53.3	52.7	10.172	387	4.988
13	23.3	23.8	23.3	23.4	0.0	0.0	53.3	52.8	10.104	467	5.067
14	23.3	23.5	23.3	23.4	23.3	23.6	30.0	29.5	10.332	332	2.766
15	43.8	43.3	8.8	9.8	8.8	8.3	38.8	38.5	10.007	376	3.531
14-3	23.3	23.0	23.3	22.7	23.3	22.3	30.0	31.9	10.008	337	2.939
16	8.8	9.0	43.8	43.9	8.8	8.4	38.8	38.6	10.832	383	3.678
17	8.8	9.3	8.8	9.6	43.8	42.8	38.8	38.2	10.195	305	3.640
18	8,8	8,9	8,8	8,8	8,8	9,0	73,8	73,3	10.075	552	7.387
19	8,8	8,4	20,4	20,0	20,4	20,2	50,4	51,4	10.255	400	4.963
20	20,4	47,1	8,8	5,7	20,4	13,7	50,4	33,5	10.746	390	4.685
14-4	23,3	24,3	23,3	22,8	23,3	22,7	30,0	30,1	10.244	330	2.760
21	20,4	19,9	20,4	8,7	8,8	21,2	50,4	50,2	10.517	392	4.828
22	20,4	20,9	20,4	21,5	20,4	20,4	38,8	37,2	10.732	360	3.496
23	26,3	25,5	26,3	26,7	8,8	9,1	38,8	38,6	10.041	404	3.667
24	26,3	25,9	8,8	9,1	26,3	26,2	38,8	38,9	10.152	357	3.500
25	26,3	25,6	8,8	8,2	8,8	8,5	56,3	57,7	10.391	440	5.553
14-5	23,3	22,4	23,3	23,5	23,3	23,7	30,0	30,5	10.179	327	2.746
26	8,8	8,6	26,3	27,2	26,3	25,9	38,8	38,3	10.043	348	3.569
27	8,8	8,5	26,3	25,9	8,8	9,0	56,3	56,5	10.610	455	5.458
28	8,8	8,7	8,8	8,9	26,3	26,0	56,3	56,4	10.163	399	5.398
29	17,5	17,9	17,5	18,0	17,5	17,4	47,5	46,7	10.196	386	4.295
14-6	23,3	23,4	23,3	24,0	23,3	22,3	30,0	30,3	10.176	329	2.791
14-7	23,3	24,0	23,3	22,6	23,3	23,1	30,0	30,3	10.029	321	2.717

Table S2. Optimization of mixed gel based on HPγCD.

Experiments	Gel1: CELLUVISC®		Gel2: GEL-LARMES®		Gel3: VISMED®		HPγCD 600mg/mL with DXMa 30 mg/mL		Adjunction of DXMa (mg)	Osmolality (mOsm/kg)	DXMa solubility (mg/mL)
	Theoretical proportion (%)	Real proportion (%)	Theoretical proportion (%)	Real proportion (%)	Theoretical proportion (%)	Real proportion (%)	Theoretical proportion (%)	Real proportion (%)			
1	70.0	70.2	0.0	0.0	0.0	0.0	30.0	29.8	5.454	382	7.510
2	0.0	0.0	70.0	70.3	0.0	0.0	30.0	29.7	5.113	230	2.804
3	0.0	0.0	0.0	0.0	70.0	69.8	30.0	30.2	5.032	239	6.223
4	0.0	0.0	0.0	0.0	0.0	0.0	100.0	100.0	5.001	796	30.448
5	35.0	34.8	35.0	35.4	0.0	0.0	30.0	29.9	5.625	367	6.972
6	35.0	35.9	0.0	0.0	35.0	34.6	30.0	29.5	5.295	290	5.866
7	35.0	35.1	0.0	0.0	0.0	0.0	65.0	64.9	5.355	326	16.978
8	0.0	0.0	35.0	35.2	35.0	34.6	30.0	30.2	5.318	260	6.640
9	0.0	0.0	35.0	36.1	0.0	0.0	65.0	63.9	2.101	502	18.965
10	0.0	0.0	0.0	0.0	35.0	37.0	65.0	63.0	5.350	381	16.727
11	0.0	0.0	23.3	23.7	23.3	23.4	53.3	52.9	5.063	370	11.688
12	23.3	24.3	0.0	0.0	23.3	23.5	53.3	52.2	5.212	304	11.079
13	23.3	23.2	23.3	23.4	0.0	0.0	53.3	53.4	5.161	441	14.819
14	23.3	23.7	23.3	23.9	23.3	22.7	30.0	29.7	5.049	313	6.771
15	43.8	43.6	8.8	9.3	8.8	9.0	38.8	38.1	5.326	284	7.963
16	8.8	8.7	43.8	43.8	8.8	9.0	38.8	38.5	5.611	351	8.886
17	8.8	9.7	8.8	8.7	43.8	44.0	38.8	37.6	5.456	296	10.039
18	8.8	8.6	8.8	10.0	8.8	9.5	73.8	71.9	5.064	475	16.334
19	8.8	8.5	20.4	20.7	20.4	19.9	50.4	50.8	5.209	362	12.604
20	20.4	20.3	8.8	9.5	20.4	20.0	50.4	50.1	5.124	286	11.972

21	20.4	21.6	20.4	19.4	8.8	9.4	50.4	49.6	5.144	407	11.885
22	20.4	21.0	20.4	20.1	20.4	19.8	38.8	39.2	5.268	324	8.571
23	26.3	26.2	26.3	26.0	8.8	9.6	38.8	38.2	5.008	341	7.706
24	26.3	26.0	8.8	9.4	26.3	26.0	38.8	38.6	5.429	284	8.550
25	26.3	26.6	8.8	9.3	8.8	8.5	56.3	55.7	5.269	446	14.230
26	8.8	9.1	26.3	26.9	26.3	26.2	38.8	37.8	5.228	324	7.088
27	8.8	8.9	26.3	26.2	8.8	9.7	56.3	55.2	2.281	405	12.869
28	8.8	9.2	8.8	9.2	26250.0	27.0	56.3	54.5	5.188	367	13.874
29	17.5	18.3	17.5	17.6	17.5	17.5	47.5	46.6	5.147	418	10.633

For both cyclodextrin derivatives, the analysis of variance (ANOVA) was carried out to determine the statistical significance of the fitted special cubic model and the coefficient terms. Basically, the values of "p-value Prob> F" less than 0.05 indicated that the selected model term were statistically significant and the values larger than 0.05 reflected that the model terms were not significant towards the output of responses [31]. All reduced models were highly significant with p-value < 0.0001 and then can be used to predict responses within the given range of factors (Table 7).

Table S3. Analysis of variance for Reduced Special Cubic Mixture models (Partial sum of squares—Type III).

		Source	Sum of square	Df	Mean square	F value	p-value Prob > F	Results
**HPβCD**	**Osmolality**	Model	2.536×10^5	6	42262.67	361.69	<0.0001	**significant**
		Linear Mixture	2.379×10^5	3	79289.97	678.57	<0.0001	
		X_1X_4	3896.27	1	3896.27	33.34	<0.0001	
		X_2X_4	3139.69	1	3139.69	26.87	<0.0001	
		X_3X_4	8777.33	1	8777.33	75.12	<0.0001	
		Residual	2570.67	22	116.85			
		Cor Total	2.561×10^5	28				
	**DXMa]**	Model	90.45	8	11.31	1239.87	<0.0001	**significant**
		Linear Mixture	89.78	3	29.93	3281.65	<0.0001	
		X_1X_4	0.13	1	0.13	14.41	0.0011	
		X_2X_3	7.392×10^{-3}	1	7.392×10^{-3}	0.81	0.3787	
		X_2X_4	0.22	1	0.22	24.58	<0.0001	
		X_3X_4	0.37	1	0.37	40.28	<0.0001	
		$X_2X_3X_4$	0.043	1	0.043	4.74	0.0416	
		Residual	0.18	20	9.119×10^{-3}			
		Cor Total	90.63	28				
**HPγCD**	**Osmolality**	Model	3.034×10^5	8	37927.80	39.40	<0.0001	**significant**
		Linear Mixture	2.307×10^5	3	76889.67	79.87	<0.0001	
		X_1X_2	2243.58	1	2243.58	2.33	0.1425	
		X_1X_4	48040.28	1	48040.28	49.90	<0.0001	
		X_2X_4	239.33	1	239.33	0.25	0.6235	
		X_3X_4	16942.75	1	16942.75	17.60	0.0004	
		$X_1X_2X_4$	5092.80	1	5092.80	5.29	0.0323	
		Residual	19252.87	20	962.64			
		Cor Total	3.227×10^5	28				
	**[DXMa]**	Model	767.80	4	191.95	102.81	<0.0001	**significant**
		Linear Mixture	761.46	3	253.82	135.94	<0.0001	
		X_1X_4	6.34	1	6.34	3.39	0.0778	
		Residual	44.81	24	1.87			
		Cor Total	812.61	28				
